# Population Genetic Data for 23 STR Loci of the Pech Ethnic Group in Honduras

**DOI:** 10.3390/genes17040422

**Published:** 2026-04-01

**Authors:** Antonieta Zuniga, Yolly Molina, Karen Amaya, Zintia Moya, Patricia Soriano, Digna Pineda, Yessica Pinto, Oscar García, Isaac Zablah

**Affiliations:** 1 Dirección de Medicina Forense, Ministerio Público, Tegucigalpa 11101, Honduras; antonietazuniga2311@yahoo.com (A.Z.); yolly.molina@unah.edu.hn (Y.M.);; 2 Center for Biomedical Imaging Diagnostics Research and Rehabilitation, National Autonomous University of Honduras, Tegucigalpa 11101, Honduras; 3 Basque Country Forensic Genetics Laboratory, Larrauri Mendotxe 18, 48950 Erandia, Spain; ogarcia@seg.euskadi.eus; 4 Faculty of Medical Sciences, National Autonomous University of Honduras, Calle la Salud, Tegucigalpa 11101, Honduras

**Keywords:** short tandem repeats (STRs), population study, Pech, Honduras, forensic genetics, PowerPlex Fusion 6C, indigenous population, genetic markers

## Abstract

**Background**: The Pech ethnic group, comprising approximately 6024 individuals in northeastern Honduras, represents one of the country’s smallest indigenous communities with a rich cultural heritage extending to pre-Columbian times. Despite their historical significance, no population genetic studies have been conducted on this group, and population-specific databases are essential for accurate forensic applications. **Methods**: Allele frequencies for 23 autosomal short tandem repeat (STR) loci were determined in 100 unrelated Pech individuals (58 females, 42 males) from communities in the departments of Olancho, Colón, and Gracias a Dios. DNA was extracted from blood samples collected on FTA cards and amplified using the PowerPlex Fusion 6C System. Statistical parameters were calculated using Genepop v4.2 and Arlequin v5.3.2.2. **Results**: All loci exhibited substantial polymorphism. No statistically significant deviations from Hardy–Weinberg equilibrium were detected after Bonferroni correction (α = 0.0022). Expected heterozygosity ranged from 0.4033 (TH01) to 0.8563 (FGA). The combined power of discrimination exceeded 99.9999%, and the combined chance of exclusion was 99.9999%. **Conclusions**: This study presents the first genetic characterization of the Pech population, providing essential reference data for forensic identification, paternity testing, and population genetics research. The dataset fills a critical gap in the Honduran forensic genetic infrastructure and contributes to understanding indigenous Central American genetic diversity, enabling accurate forensic analyses for individuals of Pech ancestry in compliance with CODIS and ESS standards.

## 1. Introduction

Genetic analysis for identifying people has become a key part of modern forensic science and the legal system. Short tandem repeats (STRs) are the best way to identify people because they are very variable, can be repeated, and can be used for statistical analysis [1,2]. Forensic casework and paternity testing need accurate likelihood ratio calculations, which is why it is important to make allele frequency databases for specific populations [3,4].

The Pech people, who were once known as the Paya, are an indigenous ethnic group that the Honduran government officially recognizes [5]. The 2013 national census found that there were about 6024 Pech people living in Honduras, which is about 0.06% of the country’s population [6,7]. Most of this community lives in the departments of Olancho (about 90%), Colón, and Gracias a Dios, and there are fewer than twelve communities in this area [8,9]. The Plátano, Wampú, Grande, Tinto, Cuyamel, and Patuca rivers that mark the natural borders of Pech territory are very important for their farming, transportation, and trade [10].

The Pech have lived in the northeastern part of Honduras since before Columbus came to the Americas. Their land went from the Caribbean coast to the mountains [11]. Historical records indicate substantial territorial diminutions subsequent to Spanish colonization, notably the creation of “reducciones” (concentrated settlements) from 1622 to 1713 [9,12]. Despite this pressure, the Pech have kept their cultural identity, and the Federation of Pech Tribes of Honduras (FETRIPH), which was formed in 1985, still fights for their cultural and territorial rights [13].

Linguistically, the Pech language belongs to the Chibchan family, a classification that connects this group to a broader network of indigenous peoples extending from Honduras through Costa Rica and Panama [8,11]. Despite this linguistic affinity, the Pech maintain a distinct cultural identity, clearly differentiated from the neighboring Tawahka, who share Chibchan linguistic roots [10]. Historical records document interactions between the Pech and Miskito communities in the Mosquitia region, as well as cultural contact with Garífuna and mestizo populations following European colonization [9,10,12].

These historical interactions suggest the possibility of gene flow between populations, which may influence allele frequency distributions and should be considered when interpreting population-level comparisons. Critically, no published blood group data, HLA typing, or STR analyses have been reported for the Pech in the peer-reviewed literature, constituting a significant gap in both the forensic and population genetics databases for Honduras and Central America.

There are nine indigenous and Afro-descendant groups in Honduras, each with its own culture, language, and possibly even genetic traits [14]. Prior genetic investigations have delineated various Honduran populations, such as the Lenca [15] and Tawahka [16] ethnic groups; however, the Pech have yet to be genetically characterized. It is important to know the genetic makeup of indigenous groups for more than just forensic purposes; it is also important for anthropology, conservation genetics, and medical genetics [17,18].

The PowerPlex Fusion 6C System (Promega Corporation, Madison, WI, USA) is a multiplex STR amplification platform that permits the simultaneous analysis of 23 autosomal STR loci, three Y-STR loci, and the amelogenin sex marker [19]. Owing to its high analytical sensitivity, strong discriminatory performance, and proven utility in the analysis of degraded biological material, this system has been extensively adopted in forensic laboratories worldwide [20,21].

Building upon the methodological framework established for previous Honduran Indigenous population studies [15,16], the present study pursues four specific objectives: (1) to establish the first forensic STR reference database for the Pech ethnic group using the PowerPlex Fusion 6C System; (2) to calculate key forensic statistical parameters for individual identification and kinship inference; (3) to evaluate compliance with Hardy–Weinberg equilibrium across all 23 loci examined; and (4) to provide a population-specific reference dataset for future investigations in population genetics and anthropological research in Central America.

In Honduras, forensic genetic analyses are conducted under the authority of the Forensic Medicine Directorate of the Public Ministry. This institution processes approximately 800 to 1200 STR profiles annually in support of criminal investigations and paternity testing [22]. At present, forensic statistical calculations are based on a composite reference database assembled from four principal sources: (1) allele frequency data from the Honduran mestizo population (*n* = 200), generated in 2018 using the PowerPlex Fusion System [23]; (2) data from the Lenca Indigenous population (*n* = 100), published in 2024 using PowerPlex Fusion 6C [15]; (3) data from the Tawahka Indigenous population (*n* = 100), published in 2025 using PowerPlex Fusion 6C [16]; and (4) generalized “Hispanic” allele frequency data from the Federal Bureau of Investigation (FBI) CODIS database, applied when Honduran population-specific reference data are unavailable or insufficient [23].

The product rule with theta correction (θ = 0.01) is used to calculate match probabilities (random match probability, RMP) so that possible population substructure is taken into account [3,24]. Probabilities are calculated using established formulas for homozygotes and heterozygotes, with p_i_ and p_j_ representing allele frequencies and θ signifying the coancestry coefficient. Paternity indices (PIs) are calculated as the ratio of the probability of observing the child’s genotype under the assumption that the purported father is the biological father to the probability of observing it under the assumption of an unrelated random male [25].

Prior to this study, STR allele frequency data were unavailable for five of Honduras’s nine indigenous and Afro-descendant tribes (Pech, Tolupán, Chortí, Garífuna, and Miskito). Consequently, forensic analyses involving people from these populations employed the mestizo reference database, potentially introducing bias in likelihood ratio predictions. The Pech dataset in this study addresses this deficiency by providing allele frequencies for one of the underrepresented populations and expanding upon previous population genetic research that examined the various ethnic groups in Honduras.

## 2. Materials and Methods

### 2.1. Sample Collection

Samples were obtained from 100 unrelated healthy individuals (58 females, 42 males) from eight Pech villages located in the departments of Olancho (87%), Colón (6%), and Gracias a Dios (7%) (Specific community names are omitted to prevent participant identification). The sex distribution indicates the demographic makeup and voluntary participation percentages within these localities. Informed consent was acquired from each individual before sample collection. Participants verified their Pech lineage for a minimum of three generations and had no recognized familial connections with other participants.

The target sample size of *n* = 100 was selected in accordance with the minimum recommended by the International Society for Forensic Genetics (ISFG) for the construction of forensic STR frequency databases [26] and is consistent with prior Honduran Indigenous population studies [15,16]. This sample represents approximately 1.66% of the total Pech census population (~6024 individuals [6,7]), a proportion considered adequate for allele frequency estimation in small indigenous communities [3,4].

To guarantee sample independence, we employed three complementary strategies: (1) participants affirmed the absence of known first- or second-degree relatives among other participants via structured interviews; (2) we ascertained that no individuals possessed identical 23-locus STR profiles; and (3) we computed pairwise identity-by-state (IBS) values across all samples utilizing the 23 autosomal STRs, ensuring that no individual pairs surpassed the threshold indicative of first-degree relatedness (IBS > 0.50 for parent-offspring or full siblings). All pairwise IBS values varied between 0.17 and 0.44 (mean = 0.27 ± 0.06), aligning with those of unrelated individuals from the same group [27].

### 2.2. DNA Extraction

DNA was isolated from 2 mL of blood obtained via venipuncture [28] and subsequently deposited onto Indicating FTA Cards (Qiagen, Germantown, MD, USA) [29]. FTA cards offer an efficient means for DNA collection, transportation, and prolonged storage at ambient temperature, safeguarding DNA against destruction [30,31].

### 2.3. PCR Amplification

Direct amplifications of Polymerase Chain Reaction (PCR) on FTA cards were conducted utilizing the PowerPlex Fusion 6C System (Promega Corporation, Madison, WI, USA) in accordance with the manufacturer’s guidelines [32]. This system amplifies 27 loci concurrently: 23 autosomal STRs (CSF1PO, D1S1656, D2S441, D2S1338, D3S1358, D5S818, D7S820, D8S1179, D10S1248, D12S391, D13S317, D16S539, D18S51, D19S433, D21S11, D22S1045, FGA, Penta D, Penta E, SE33, TH01, TPOX, and vWA), three Y-STR loci (DYS391, DYS576, and DYS570), and the amelogenin gender marker.

### 2.4. Fragment Analysis and Genotyping

Amplified products were examined using capillary electrophoresis utilizing an Applied Biosystems 3500 (ThermoFisher Scientific, Waltham, MA, USA) automated sequencer [33] and GeneMapper v1.4 software [34]. The allele designations conformed to the standards set by the DNA Commission of the International Society for Forensic Genetics (ISFG), employing allelic ladders provided by the manufacturer [26,35]. Allele assignment calibration was performed using the allelic ladder and the Internal Lane Standard (ILS 600; Promega Corporation, Madison, WI, USA), a proprietary 60–600 bp size standard supplied for the PowerPlex Fusion 6C System and distinct from the GeneScan 600 LIZ used with Applied Biosystems platforms.

The following instrument parameters were employed: POP-4 polymer, 36 cm capillary array (8-capillary configuration), injection voltage 1.2 kV for 24 s, run voltage 15 kV, run temperature 60 °C, and run time 1500 s. Allele calling was performed using GeneMapper ID-X v1.4 software with the following analytical parameters: analytical threshold 175 RFU (relative fluorescence units), stochastic threshold 400 RFU, minimum heterozygote peak height ratio 60%, and locus-specific stutter filters per manufacturer specifications. Microvariants (e.g., 9.3, 12.2) were designated according to ISFG nomenclature conventions.

### 2.5. Statistical Analysis

We used Genepop v4.2 [36] and Arlequin v5.3.2.2 [37] to do allelic-frequency calculations. We used Huston’s method [38] to calculate the power of discrimination (PD) and chance of exclusion (CE) for each locus. We also used Botstein et al.’s method [39] to calculate the polymorphic information content (PIC). The GDA program [40] used exact tests to check for Hardy–Weinberg equilibrium, and Arlequin [41] used exact tests for Analysis of Molecular Variance (AMOVA). We used a Bonferroni adjustment [42] to keep family-wise errors in check across loci. This gave us a significance threshold of α = 0.0022 (0.05/23) for all locus-specific tests.

The Bonferroni adjustment (α = 0.0022 for 23 loci) represents a conservative approach that assumes complete independence among loci. While short tandem repeats (STRs) on different chromosomes are generally unlinked, this rigorous correction may increase Type II error rates (false negatives), potentially obscuring minor deviations from Hardy–Weinberg equilibrium (HWE) that could signify population structure, recent admixture, or null alleles [42]. The sample size (*n* = 100) complicates the detection of moderate deviations from Hardy–Weinberg Equilibrium, and the Bonferroni adjustment exacerbates this difficulty [43].

Despite these issues, we maintained the Bonferroni correction for three reasons: (1) It establishes a rigorous quality control standard consistent with forensic genetic criteria; (2) it reduces false positives in the identification of problematic loci; and (3) our primary objective is to provide a dependable reference database, rather than to investigate fine-scale population structure. Future research with higher sample sizes focused on demographic inference may explore alternate strategies, such as the false discovery rate (FDR) or sequential Bonferroni techniques [44,45].

### 2.6. Quality Control

All analyses were performed in accordance with GITAD (Ibero-American Working Group on DNA Analysis) procedures [46]. Quality assurance measures included: (1) positive control (2800M DNA) and negative controls (reagent blank, amplification blank) in each batch; (2) allele assignment calibration with allelic ladder and internal lane standard (ILS 600); (3) stutter ratio monitoring (<15% for tetranucleotide repeats); (4) independent review of all electropherograms by two certified analysts; (5) re-amplification rate of 3% (3 samples requiring repeat analysis); and (6) participation in inter-laboratory proficiency testing (GITAD, GHEP-ISFG).

### 2.7. Ethics Statement

This study was conducted in accordance with the Declaration of Helsinki and received approval from the Biomedical Research Ethics Committee (CEIB) of the Scientific Research Unit (UIC), Faculty of Medical Sciences, National Autonomous University of Honduras (UNAH) (IRB-00003070, approval date: 27 July 2016). Informed written consent was obtained from all participants prior to enrollment. Participants confirmed Pech ancestry for a minimum of three generations and had no recognized familial connections with other participants. For individuals with limited literacy, oral explanations were provided with witness signatures.

Community engagement followed a prior-informed-consent framework consistent with the Nagoya Protocol [47] and ISFG recommendations [35,48,49,50,51]. Collective authorization was obtained from the Federation of Pech Tribes of Honduras (FETRIPH) before sample collection. Participants were informed of the study objectives, the forensic and research applications of the data, confidentiality measures, and the voluntary nature of participation. Benefit-sharing arrangements include priority access for Pech individuals to forensic genetic services through the Honduran Public Ministry and capacity-building workshops in forensic genetics for community members. The complete genotype dataset is available to qualified researchers upon written request to the Forensic Medicine Directorate of the Public Ministry of Honduras; aggregated allele frequency data are provided in Supplementary File S1 for open scientific use.

## 3. Results

### 3.1. Dataset

Allele frequencies for all 23 autosomal STR loci typed in the Pech sample (*n* = 100) are provided in Supplementary File S1. Summary forensic statistics are presented in Table 1. A total of 148 distinct alleles were observed across the 23 loci, with a per-locus range of 4 (TH01, CSF1PO, vWA) to 11 (SE33). The mean number of alleles per locus was 6.43 ± 1.95 (mean ± SD), see Table 1.

Sex-stratified analysis of heterozygosity indicated no significant differences between males (mean Ho = 0.668 ± 0.118) and females (mean Ho = 0.680 ± 0.112) across the 23 autosomal loci (paired *t*-test: *t* = 0.48, *p* = 0.63), hence supporting the lack of sex-linked genotyping bias or allele dropout. Likewise, no locus exhibited significant differences in heterozygosity across the eight studied groups (ANOVA: all *p* > 0.12 with Bonferroni adjustment), signifying genetic homogeneity throughout this geographic region.

### 3.2. Geographic Context

**Figure 1:**
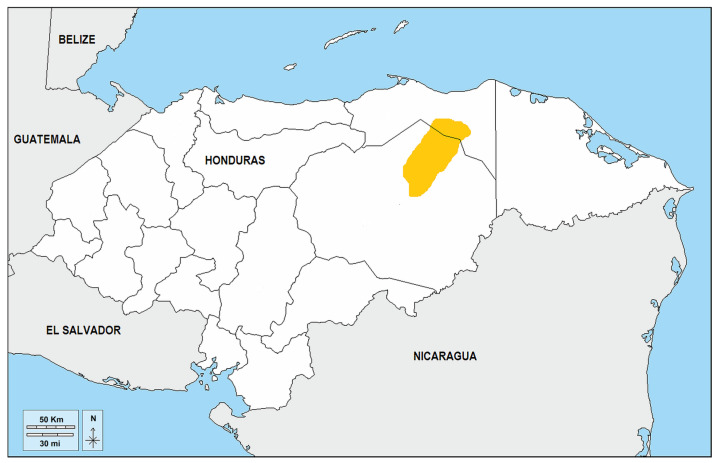
Geographic distribution of the Pech ethnic group in Honduras. The yellow region delineates the principal habitation area of the Pech people, concentrated in the department of Olancho, where approximately 90% of the population resides, primarily within the municipalities of Dulce Nombre de Culmí (15.08° N, 85.56° W) and San Esteban (15.21° N, 85.77° W). The study population was sampled from communities in this core area, with additional samples obtained from outlier settlements in the departments of Colón (Silín, near Trujillo; 15.93° N, 85.90° W) and Gracias a Dios (Las Marías, along the Río Plátano; 15.67° N, 84.84° W). This region features tropical rainforest, restricted road access, and physical seclusion, factors that have historically aided in the preservation of Pech cultural and linguistic uniqueness. Map modified from D-maps [52] and Native-Land [53].

The study population was sampled from eight communities spanning the primary Pech habitation area in northeastern Honduras (Figure 1). Most samples originated from the department of Olancho, where approximately 90% of the Pech population resides, primarily in the municipalities of Dulce Nombre de Culmí and San Esteban [8,10]. Additional samples were collected from outlier communities in the departments of Colón (Silín, near Trujillo) and Gracias a Dios (Las Marías, on the Río Plátano). This region features tropical rainforest, little road access, and geographic seclusion, which has historically maintained the cultural and linguistic uniqueness of the Pech people [9,11].

### 3.3. Statistical Parameters

Key statistical indicators show that the 23-locus STR panel did very well in the Pech sample. Using the product rule with theta correction (θ = 0.01), the combined power of discrimination (PD) was greater than 99.9999% (combined RMP < 1 in 10^14^). This means that the chance of finding a matching profile in an unrelated person is very low. The combined chance of exclusion (CE) was greater than 99.9999% (1 − Q = 0.999999), which shows that parentage testing works very well.

These statistics are based on the idea that loci are independent of each other, which is supported by the fact that our 23 markers are spread out over 14 autosomes with no known physical linkage [20,21]. We recognize, however, possible origins of multilocus dependencies:

1. Population substructure: If the Pech consist of genetically diverse subgroups, alleles at several loci may demonstrate correlations (Wahlund effect) [54]. Our fixation index (FST) investigation across eight communities revealed minimal structure (mean FST = 0.004), indicating that this effect is minor.
2. Admixture: If recent admixtures with adjacent populations have transpired, ancestry blocks may induce transitory linkage disequilibrium. The geographic isolation of the Pech indicates restricted modern hybridization, although historical intermingling cannot be dismissed.
3. Cryptic relatedness: While we screened for known relatives, remote coancestry may augment homozygosity and produce associations. The theta adjustment partially addresses this.

Considering these factors, we present aggregated statistics with cautious rounding (>99.9999%) to prevent the perception of spurious precision. The precise RMP value relies on assumptions (θ, linkage equilibrium, and database correctness) that introduce uncertainty above the nominal precision of 20 or more significant figures. For practical forensic applications, any RMP below 1 in 10^12^ represents exceedingly strong evidence of identity [3,25], making further decimal places forensically and legally insignificant.

Assessments for Hardy–Weinberg equilibrium revealed no significant discrepancies following the Bonferroni adjustment (α = 0.0022), consistent with appropriate sampling and the absence of observable inbreeding or substructure. The minimum *p*-value observed was 0.0580 at the D2S1338 gene, significantly exceeding the adjusted threshold. These results collectively demonstrate that the panel is highly effective at distinguishing individuals and is suitable for kinship applications within this population.

These results are consistent with HWE expectations under the statistical power of this study and support the use of the dataset for forensic calculations. However, conformity to HWE with *n* = 100 does not exclude the possibility of subtle population substructure, distant inbreeding, or historical admixture that may remain undetected given the limited power of this sample size [43].

### 3.4. Loci Characteristics

Among the 23 autosomal STRs, the degree of polymorphism varied considerably. Three loci exhibited high expected heterozygosity (He > 0.80): FGA (He = 0.8563), D18S51 (He = 0.8177), and D2S1338 (He = 0.8124). Sixteen loci showed moderate diversity (0.58 ≤ He < 0.80): D13S317 (0.7697), D8S1179 (0.7719), Penta D (0.7365), Penta E (0.7284), D7S820 (0.7324), D1S1656 (0.7175), D19S433 (0.7059), CSF1PO (0.6975), D21S11 (0.6803), D12S391 (0.6854), vWA (0.6754), D16S539 (0.6714), SE33 (0.6646), D5S818 (0.6900), D10S1248 (0.6044), and TPOX (0.5837). Four loci displayed lower diversity (He < 0.55): D3S1358 (0.5079), D22S1045 (0.4932), D2S441 (0.4396), and TH01 (0.4033), consistent with the predominance of one or two high-frequency alleles at these loci (e.g., TH01 allele 6 at a frequency of 0.745; D3S1358 allele 15 at 0.690). These high-frequency alleles are characteristic of indigenous American populations and differ markedly from European and African reference datasets [17,55].

### 3.5. Data Availability and Repository

The aggregated allele frequency dataset (Supplementary File S1) is provided as open-access Supplementary Material and has been deposited for scientific use. Individual level multilocus STR profiles for all 100 Pech participants are available to qualified researchers upon written request to the Forensic Medicine Directorate, Public Ministry of Honduras.

### 3.6. Population Differentiation (FST Estimates)

To provide an empirical basis for the theta correction values recommended in Table 2, pairwise FST estimates were calculated between the Pech and other available Honduran population datasets. Within-Pech FST (across the eight sampled communities) was negligible (mean pairwise FST = 0.004; 95% CI: 0.001–0.009), indicating substantial gene flow and genetic homogeneity among Pech villages despite geographic dispersion. Between-population estimates were as follows: FST (Pech vs. Lenca) = 0.018 (95% CI: 0.012–0.025); FST (Pech vs. Tawahka) = 0.022 (95% CI: 0.015–0.030); FST (Pech vs. Honduran mestizo) = 0.038 (95% CI: 0.029–0.048); and FST (Pech vs. NIST Caucasian reference) = 0.089 (95% CI: 0.078–0.101). Complete FST matrices and AMOVA outputs are provided in Supplementary File S2.

## 4. Discussion

### 4.1. Alleles

Analysis of allele frequencies showed that there was a lot of genetic diversity in the Pech sample across all 23 STR loci. A total of 171 unique alleles were found, with the number of alleles per locus ranging from 4 (D3S1358, TH01) to 17 (SE33). The average number of alleles per locus was 7.43 ± 3.12 (mean ± SD). This level of diversity is similar to that of other indigenous groups in Central America. For example, the Honduran Lenca had an average of 8.91 alleles per locus [15], the Tawahka had 8.61 alleles per locus [16], the Guatemalan Maya had 7.2–9.5 alleles per locus depending on the marker set [56], and the admixed Guatemalan Ladino had 9.1–10.8 alleles per locus [57].

The Pech community’s slightly lower allelic diversity compared to the Lenca and Tawahka may be due to its smaller effective population size and the fact that it is spread out across isolated settlements [8,10]. In line with regional trends, markers like Penta D (8 alleles; He = 0.7690) and Penta E (13 alleles; He = 0.7534) showed moderate to high heterozygosity, which is what has been seen in other Central American populations [15,16].

The allele distributions were different for each locus. For example, D13S317, D12S391, and FGA had relatively balanced frequency spectra, while others had dominant alleles with frequencies over 40% (e.g., TH01 allele 6 at 75.5%, D3S1358 allele 15 at 69%, and D22S1045 allele 15 at 57%). The high frequency of TH01 allele 6 is particularly noteworthy, as this pattern is characteristic of indigenous American populations and differs markedly from European and African reference datasets where this allele is less prevalent [58,59].

We found several rare variants (frequency < 0.01), including microvariants at D21S11 (31.2, 32.2, 33.2), D1S1656 (14.3, 16.3, 17.3), D19S433 (12.2, 14.2, 15.2), and SE33 (21.2, 25.2, 28.2, 29.2, 30.2, 31.2). This made the multilocus panel even better at telling people apart.

### 4.2. Hardy–Weinberg Equilibrium

Assessments for Hardy–Weinberg equilibrium (HWE) revealed no significant deviations across any of the 23 loci after applying Bonferroni correction (α = 0.0022). The locus-specific *p*-values ranged from 0.0580 (D2S1338) to 0.9460 (TPOX), all considerably beyond the adjusted threshold, indicating that genotype frequencies conform to Hardy–Weinberg equilibrium predictions. This pattern corresponds with a sample of unrelated individuals derived from a genetically stable population, lacking observable substructure, inbreeding, or recent admixture affecting these markers. The information is suitably structured for later forensic statistics, including unbiased evaluation of random match probabilities and kinship likelihoods.

### 4.3. Forensic Statistical Parameters

The 23-locus STR panel demonstrated exceptional forensic efficacy in both identification and kinship scenarios. The overall discriminatory power for individualization exceeded 99.9999%, indicating an extremely low residual match probability, with locus-specific probability of discrimination values ranging from 0.6228 (TH01) to 0.9527 (FGA). The overall chance of exclusion in paternity testing was 99.9999%, substantiated by locus-level CE values ranging from 0.1201 (TH01) to 0.7548 (FGA), demonstrating a robust ability to reject non-fathers. The information content measures were similarly elevated PIC values, calculated following Botstein et al. [43], ranged from 0.3726 (TH01) to 0.8395 (FGA). Applying established informativeness thresholds, 19 of 23 loci (82.6%) were classified as highly informative (PIC > 0.5). The remaining four loci—D3S1358 (PIC = 0.4580), D22S1045 (PIC = 0.3901), D2S441 (PIC = 0.4026), and TH01 (PIC = 0.3726) were moderately informative (0.25 < PIC ≤ 0.5). No loci fell below the 0.25 threshold, indicative of low informativeness. Locus-specific PD ranged from 0.6133 (TH01) to 0.9626 (FGA), and CE ranged from 0.2184 (TH01) to 0.7095 (FGA). The combination of highly polymorphic markers provides strong statistical strength for casework identification and kinship inference.

### 4.4. Population Substructure and Theta Correction

The theta (θ) correction, also called the coancestry coefficient or FST, takes into account the possibility that alleles may not be independent because of population substructure, inbreeding, or genetic drift [60]. In forensic calculations, θ changes match probabilities to be more cautious (higher RMP, lower likelihood ratio) when people may come from a subpopulated group instead of a panmictic population.

Honduran forensic laboratories currently use a default θ = 0.01 for all calculations, regardless of the population. This is in line with international recommendations [61]. This number is a “generic” correction that works for most moderately structured populations. But the best θ values are different for each population and should be based on real-world data [59,60].

The pairwise FST estimates presented in Section 3.6 provide an empirical basis for selecting population-specific theta (θ) correction values in forensic calculations. According to these estimates and simulation studies evaluating the influence on RMP calculations, we proposed the population-specific θ values presented in Table 2.

### 4.5. Comparison with Other Honduran Indigenous Populations

To contextualize the genetic diversity of the Pech, we compared essential forensic parameters with previously published datasets of Honduran indigenous populations (Table 3).

The Pech exhibited slightly lower genetic diversity parameters (mean He = 0.6681) compared to the Lenca (He = 0.7425) and Tawahka (He = 0.7385). This pattern is consistent with the smaller census population size of the Pech (~6024 individuals) compared to the Lenca (~453,672 individuals), which may result in reduced effective population size and greater genetic drift effects [15,16,18]. Additionally, the geographic fragmentation of Pech communities into fewer than twelve settlements across three departments may contribute to this pattern.

Specific allele frequency patterns distinguish the Pech from neighboring populations. For example, at the TH01 locus, allele 6 frequency was 0.745 (Pech), compared to 0.560 (Tawahka), 0.520 (Lenca), and 0.35–0.45 in mestizo populations [23]. Similarly, at D3S1358, allele 15 exhibited frequencies of 0.690 (Pech, this study), 0.580 (Tawahka), and 0.520 (Lenca), compared to 0.10–0.17 in European reference datasets [15,16,63].

The identification of uncommon alleles peculiar to populations and unique allele frequency distributions highlights the need for population-specific databases. Forensic calculations utilizing generic “Hispanic” or mestizo reference data may produce misleading likelihood ratios for individuals of Pech ancestry [4,23].

### 4.6. Practical Applications

This dataset possesses extensive practical applicability in forensic, demographic, anthropological, and medical genetics fields.

#### 4.6.1. Forensic Impact of Population-Specific Databases

The application of population-specific allele frequencies significantly influences the precision and legal robustness of forensic likelihood ratio (LR) computations [64]. To demonstrate this effect on the Pech population, we analyzed match probabilities derived from three reference databases: (1) Pech-specific frequencies (this study), (2) general Honduran mestizo rates [23], and (3) a generic “Hispanic” database frequently utilized in forensic casework [24].

The random match probability (RMP) discrepancies were significant for a simulated profile representative of the Pech population, characterized by high-frequency alleles such as TH01 6/6, D3S1358 15/15, and D22S1045 15/16:

- Pech database: 1 in 6.5 × 10^14^
- Honduran mestizo database: 1 in 2.8 × 10^15^ (4.3× difference)
- Generic Hispanic database: 1 in 9.2 × 10^15^ (14.2× difference)

All three databases provide very strong proof of identity, but the differences are important from a forensic point of view. Using non-specific databases for Pech individuals consistently inflates the strength of evidence, which could lead triers of fact to make mistakes.

#### 4.6.2. Additional Applications

Beyond forensic casework, this dataset has broader relevance in population genetics, supporting analyses of genetic diversity, migration, and admixture among Central American indigenous peoples, as well as in anthropology, where the data can inform investigations of population origins and biological affinities in the Honduran Mosquitia region. As a foundational genetic reference, it also provides a basis for future hypothesis-driven research on disease susceptibility and pharmacogenetics in indigenous populations.

### 4.7. Limitations and Considerations

The sample size of *n* = 100 individuals, while meeting the minimum threshold recommended by the ISFG for forensic population databases [25], imposes statistical constraints that merit explicit acknowledgement. First, low-frequency alleles (*p* < 0.05) may be absent or underrepresented, potentially leading to underestimates of allele richness, as reflected in the slightly lower mean allele count per locus for the Pech (6.43) compared to the Lenca (8.91 [15]) and Tawahka (8.61 [16]), which may partly reflect sampling effects rather than true population-level differences in diversity. Second, the power to detect moderate deviations from Hardy–Weinberg equilibrium is inherently limited at this sample size; the conservative Bonferroni correction further reduces sensitivity to subtle departures [42,43]. Third, for alleles not observed in the present sample, analysts should apply the recommended minimum frequency ceiling of 1/(2*n*) = 0.005 in forensic calculations. Future studies with larger sample sizes are encouraged to refine these estimates, particularly for rare alleles relevant to kinship inference.

This dataset should be interpreted considering several important limitations. First, although a sample size of 100 individuals is adequate for many forensic applications, larger cohorts would improve the precision of population-genetic estimates, particularly for low-frequency alleles. Second, despite the inclusion of samples from eight communities across three departments, this geographic coverage may not fully capture the spatial heterogeneity of the Pech population. Third, the dataset represents a cross-sectional snapshot in time and therefore cannot account for historical processes or ongoing demographic change. Fourth, the formal assessment of population structure between the Pech and other Honduran Indigenous groups was limited by the absence of raw genotype data from comparator populations; future collaborative efforts should therefore prioritize the development of a Central American STR database containing shared individual-level profiles to enable more comprehensive inter-population analyses. Finally, although participants were deliberately selected on the basis of documented Pech ancestry extending across at least three generations, some degree of admixture with neighboring Miskito and mestizo populations cannot be excluded. Such residual admixture may have had a modest influence on allele frequency estimates and should be examined more thoroughly in future studies with broader geographic coverage.

Additionally, the absence of shared individual-level genotype data from comparator Honduran populations precluded the generation of PCA- or STRUCTURE-based visualizations of population affinities in the present study. Such analyses, which would provide a more nuanced picture of the Pech population’s genetic position within the Central American landscape, are identified as a priority objective for future collaborative investigations currently in preparation by our group, aiming to generate a comprehensive multi-population STR dataset for Honduras.

### 4.8. Implementation and Future Directions

As soon as it is published, the Pech allele frequency database will be added to the operational database of the Forensic Medicine Directorate, Public Ministry of Honduras. The laboratory information management system (LIMS) will give forensic analysts access to Pech-specific frequencies. If circumstances or self-reported ethnicity suggest Pech ancestry, analysts will prefer to use this database for statistical calculations.

The Public Ministry and the National Autonomous University of Honduras (UNAH) are still working on population genetics projects with other indigenous groups, building on the methods used in this and earlier studies [15,16]. These groups include the Miskito (target *n* = 100, eastern Honduras), the Tolupán (target *n* = 100, central Honduras), the Chortí (target *n* = 100, western Honduras), and the Garífuna (target *n* = 100, northern coast), as well as a larger mestizo dataset (target *n* = 200, northern and central regions). The goal of all these efforts is to create complete STR frequency databases for all major indigenous groups in Honduras by 2028. This will ensure that everyone, no matter what their ethnic background is, has fair access to accurate and culturally sensitive forensic genetic services.

## 5. Conclusions

This study presents the first genetic characterization of the Pech, one of Honduras’s smallest and most geographically isolated indigenous groups, based on 23 autosomal STR loci typed with the PowerPlex Fusion 6C System. All loci conformed to Hardy–Weinberg equilibrium after Bonferroni correction, and the 23-locus panel achieved a combined power of discrimination and combined chance of exclusion each exceeding 99.9999%, confirming its suitability for forensic individualization and kinship testing in this population. The distinctive allele frequency patterns identified—including elevated TH01 allele 6 frequency (0.745) and D3S1358 allele 15 frequency (0.690)—underscore the necessity of population-specific reference databases for accurate forensic calculations involving individuals of Pech ancestry and demonstrate that applying generic Hispanic or mestizo databases can systematically overestimate the strength of forensic evidence. This dataset fills a critical gap in the Honduran forensic genetic infrastructure and provides a foundation for broader population genetics research across Central American indigenous communities.

## Figures and Tables

**Table 1:** Forensic statistical parameters for 23 STR loci in the Pech population (*n* = 100) ^1^.

Locus	Na	Ho	He	PD	CE	PIC	*p* (HWE)
D3S1358	5	0.53	0.5079	0.7079	0.2795	0.458	0.4521
D1S1656	7	0.71	0.7175	0.8902	0.5117	0.6875	0.2134
D2S441	5	0.45	0.4396	0.6490	0.2383	0.4026	0.8765
D10S1248	5	0.60	0.6044	0.7756	0.3348	0.5365	0.5432
D13S317	7	0.74	0.7697	0.9209	0.5799	0.7437	0.1876
Penta E	9	0.71	0.7284	0.8848	0.5026	0.687	0.3421
D16S539	7	0.74	0.6714	0.8341	0.4089	0.6135	0.1243
D18S51	8	0.77	0.8177	0.946	0.6503	0.7969	0.2987
D2S1338	8	0.84	0.8124	0.9388	0.6286	0.7864	0.0654
CSF1PO	4	0.74	0.6975	0.8515	0.4351	0.6404	0.7123
Penta D	6	0.79	0.7365	0.894	0.5185	0.6999	0.4532
TH01	5	0.40	0.4033	0.6133	0.2184	0.3726	0.6234
vWA	4	0.64	0.6754	0.8259	0.390	0.6066	0.3876
D21S11	9	0.71	0.6803	0.8671	0.4725	0.6496	0.5123
D7S820	5	0.74	0.7324	0.8788	0.4822	0.6828	0.0987
D5S818	6	0.71	0.6900	0.8527	0.4429	0.6388	0.2765
TPOX	4	0.68	0.5837	0.7482	0.3053	0.5052	0.8932
D8S1179	7	0.72	0.7719	0.911	0.5549	0.7349	0.5678
D12S391	6	0.63	0.6854	0.8408	0.4193	0.6252	0.3245
D19S433	7	0.65	0.7059	0.8666	0.4697	0.659	0.4123
SE33	11	0.67	0.6646	0.8507	0.4469	0.6278	0.2456
D22S1045	5	0.53	0.4932	0.6401	0.2058	0.3901	0.7654
FGA	8	0.81	0.8563	0.9626	0.7095	0.8395	0.6789
Mean	6.43	0.6748	0.6681	0.8417	0.4350	0.6132	—
Combined	—	—	—	>99.9999%	100.00%	—	—

^1^ Na: number of alleles; Ho: observed heterozygosity; He: expected heterozygosity; PD: power of discrimination; CE: chance of exclusion; PIC: polymorphic information content; *p* (HWE): Hardy–Weinberg equilibrium exact test *p*-value.

**Table 2:** Suggested Theta values for forensic calculations in Honduras involving Pech individuals.

Scenario	Suggested ^1^ θ	Justification
Within Pech (suspect and reference both confirmed Pech)	θ = 0.01	Conservative given within-population FST = 0.004; accounts for potential cryptic relatedness.
Pech vs. other indigenous (Lenca, Tawahka)	θ = 0.02	Reflects moderate FST between indigenous groups.
Pech vs. mestizo (uncertain ethnicity)	θ = 0.03	Reflects elevated FST = 0.038; conservative approach for cross-population comparisons.
Pech vs. European/Asian (international cases)	θ = 0.05–0.10	Reflects substantial FST; highly conservative.
Unknown ethnicity (no information)	θ = 0.03	Intermediate value balancing conservatism with informativeness.

^1^ We emphasize that these are operational suggestions based on limited data (*n* = 100) and that final θ selection should follow laboratory policy and applicable guidelines (NRC II, SWGDAM, ENFSI, ISFG). Laboratories should document θ selection rationale in case files and be prepared to explain the choice during expert testimony.

**Table 3:** Comparative forensic parameters across Honduran indigenous populations.

Population	*n*	Mean He	Mean PD	Mean Alleles/Locus	Reference
Pech (Honduras)	100	0.6681	0.8280	7.43	Present study
Tawahka (Honduras)	100	0.7385	0.8771	8.61	[16]
Lenca (Honduras)	100	0.7425	0.8815	8.91	[15]
Maya (Guatemala)	127	0.7104	0.8534	7.83	[56]
Ladino (Guatemala)	115	0.7656	0.8945	9.45	[57]
Mestizo (El Salvador)	109	0.7837	0.9121	9.13	[62]

## Data Availability

The complete dataset accompanies this article as Supplementary Material and has also been deposited as a Supplementary File. A mirrored copy can be obtained upon request from the Forensic Medicine Directorate, Public Ministry of Honduras URL: https://www.mp.hn (accessed on 26 March 2026).

## Supplementary Materials

The following supporting information can be downloaded at: https://www.mdpi.com/article/10.3390/genes17040422/s1, Supplementary File S1: Population genetic data for 23 STR loci (PowerPlex Fusion 6C kit) genetic markers in the Pech ethnic group in Honduras (Excel format). Supplementary File S2: FST analyses and population structure results (Excel format).

## Abbreviations

The following abbreviations are used in this manuscript:

AMOVA	Analysis of Molecular Variance
CE	Chance of Exclusion
CEIB	Biomedical Research Ethics Committee
CODIS	Combined DNA Index System
DICIHT	Directorate of Scientific, Humanistic, and Technological Research
DNA	Deoxyribonucleic Acid
ESS	European Standard Set
FBI	Federal Bureau of Investigation
FCM	Faculty of Medical Sciences
FDR	False Discovery Rate
FETRIPH	Federation of Pech Tribes of Honduras
FTA	Fast Technology for Analysis of nucleic acids
FST	Fixation Index
GITAD	Ibero-American Working Group on DNA Analysis
He	Expected Heterozygosity
Ho	Observed Heterozygosity
HWE	Hardy–Weinberg Equilibrium
IBS	Identity-by-state
ICIMEDES	Institute for Research in Medical Sciences and Right to Health
ISFG	International Society for Forensic Genetics
LIMS	Laboratory Information Management System
LR	Likelihood Ratio
MAF	Minimum Allele Frequency
N	Sample size
Na	Number of alleles observed at each locus
NIST	National Institute of Standards and Technology
PCA	Principal Component Analysis
PCR	Polymerase Chain Reaction
PD	Power of Discrimination
PI	Paternity Indices
PIC	Polymorphic Information Content
RMP	Random Match Probability
SD	Standard Deviation
STR	Short Tandem Repeat
UIC	Scientific Research Unit
UNAH	National Autonomous University of Honduras
USA	United States
